# High-Flow-Rate Trace Formaldehyde Detection Based on Ultraviolet Photoacoustic Spectroscopy Using a Long Resonant Photoacoustic Cell

**DOI:** 10.3390/s26051410

**Published:** 2026-02-24

**Authors:** Qianjin Gan, Zhongqi Feng, Deng Zhang, Shibang Ma, Xiu Yang, Xukun Yin

**Affiliations:** 1 School of Optoelectronic Engineering, Xidian University, Xi’an 710071, China; 2 Xi’an Institute of Applied Optics, Xi’an 710065, China

**Keywords:** formaldehyde detection, ultraviolet photoacoustic spectroscopy, high gas flow rate

## Abstract

Formaldehyde (H_2_CO) is a hazardous volatile organic compound widely present in indoor and industrial environments, and its real-time, highly sensitive detection is essential for environmental safety. However, existing detection techniques often face challenges in simultaneously achieving high sensitivity and long-term stability, and many conventional photoacoustic spectroscopy (PAS) systems rely strongly on low gas flow rates to suppress flow-induced noise, which limits their applicability for continuous online monitoring. In this work, an ultraviolet photoacoustic spectroscopy (UV-PAS)-based H_2_CO detection system operating in a nitrogen (N_2_) background is developed. The system integrates a compact differential photoacoustic cell (PAC) with a 320 nm ultraviolet laser source, in which the resonator length and buffer configuration are carefully optimized to enhance acoustic resonance and effectively suppress flow-related disturbances. Notably, a key innovation of this study is that the system maintains a stable photoacoustic response even under relatively high gas flow conditions. Experimental results demonstrate that at a flow rate of 250 sccm, the photoacoustic signal amplitude remains stable, and the noise level is well controlled, significantly reducing the dependence of conventional PAS systems on low-flow operation. The photoacoustic cell exhibits a resonant frequency of 1767 Hz and a quality factor of 46. Calibration using a 47.31 ppm H_2_CO:N_2_ gas mixture shows a good linear response with a correlation coefficient of R^2^ = 0.98844. The minimum detection limit reaches 2.50 ppm at a 1 s integration time and is further improved to 88.1 ppb at an integration time of 2202 s based on Allan–Werle deviation analysis. These results demonstrate that the proposed UV-PAS system provides a sensitive, stable, and cost-effective solution for real-time trace H_2_CO detection while retaining robust performance at elevated gas flow rates, highlighting its strong potential for practical applications.

## 1. Introduction

Formaldehyde (H_2_CO) is a critical pollutant in indoor air, industrial exhaust, and environmental photochemistry, where it plays a central role in atmospheric oxidation pathways, secondary organic aerosol formation, and indoor air-quality degradation [1]. Due to its high volatility and strong irritant properties, even trace concentrations of H_2_CO can adversely affect human health. Numerous epidemiological studies have linked chronic exposure to formaldehyde with increased risks of respiratory inflammation, asthma, reduced lung function, and nasopharyngeal cancer [2,3,4,5]. Consequently, international regulatory agencies, including the U.S. Environmental Protection Agency (EPA) and the World Health Organization (WHO), have established stringent exposure limits, thereby driving the development of detection technologies capable of quantifying H_2_CO at sub-ppm or even ppb levels [6].

Laser-based spectroscopic techniques, including ultraviolet absorption, mid-infrared (MIR) absorption, cavity-enhanced absorption spectroscopy (CEAS), and photoacoustic spectroscopy, have therefore become major research directions for real-time formaldehyde sensing [7]. UV differential absorption and CEAS-based systems have demonstrated sub-ppb sensitivity for indoor air monitoring by exploiting the strong UV absorption features of H_2_CO and extended effective optical path lengths [8,9,10,11]. In parallel, chromatographic, spectrophotometric, colorimetric, and electrochemical methods have been widely explored for environmental, food-safety, and consumer-product analysis, offering complementary advantages in chemical specificity and deployment flexibility [12,13,14,15,16,17,18,19].

In the recent years, substantial progress has been achieved worldwide in PAS-based gas detection technologies, particularly for trace and ultra-trace species [20,21]. Differential photoacoustic cells, cantilever-enhanced PAS (CE-PAS), and quartz-enhanced PAS (QEPAS) have enabled ppb- to ppt-level detection of gases such as formaldehyde, nitrogen dioxide, sulfur dioxide, methane, and SF_6_ decomposition products [22,23,24,25,26,27,28,29,30].In parallel, tunable diode laser absorption spectroscopy (TDLAS) offers high sensitivity and selectivity through extended optical path lengths and advanced signal processing, enabling ppb-level detection of trace gases such as HF and CH_4_. In addition, recent cavity-enhanced Raman spectroscopy (CERS) implementations, including parabolic-mirror and circular cavity designs, demonstrate that optimized optical confinement can significantly strengthen light–matter interaction for sensitive trace gas detection [31,32,33,34,35].Notably, compact differential photoacoustic cells (PACs), including 3D-printed implementations, have achieved ppb-level H_2_CO detection using low-power ultraviolet laser sources. Meanwhile, cantilever-enhanced photoacoustic spectroscopy (CE-PAS) and quartz-enhanced photoacoustic spectroscopy (QEPAS) systems, employing cantilevers and quartz tuning forks as acoustic wave detectors, respectively, have realized highly sensitive detection of other trace gases under excitation by quantum cascade lasers or interband cascade lasers, demonstrating pronounced advantages in both sensitivity and stability [36,37,38,39,40]. More recently, light-induced thermoelastic spectroscopy (LITES), which exploits thermoelastic vibration of quartz or lithium niobate tuning forks under optical excitation, has emerged as a powerful extension of PAS, enabling high-frequency operation, calibration-free absolute measurements, and ppb-level sensitivity with enhanced immunity to acoustic and flow-induced noise [41,42,43]. Compared with QEPAS- and LITES-based systems that rely on tuning-fork transducers operating at ultrasonic frequencies, the present UV-PAS system adopts a resonant differential photoacoustic cell optimized for stable standing-wave formation, offering improved tolerance to elevated gas flow rates and robust signal stability under continuous-flow conditions. These advances highlight the potential of optimized PAS architectures to achieve high sensitivity while maintaining low size, weight, and power consumption.

Photoacoustic spectroscopy operates by converting modulated optical absorption into periodic pressure waves through rapid non-radiative relaxation processes. The resulting acoustic amplitude scales linearly with the absorption coefficient, laser power, and molecular concentration, providing a direct and background-free measurement of trace gases. Compared with infrared implementations, ultraviolet PAS offers distinct advantages, such as higher photon energy promotes efficient electronic excitation and non-radiative relaxation, while spectral interference from water vapor and other atmospheric constituents is significantly reduced in the UV region [44,45,46,47,48,49]. These features enable stronger signal generation, improved selectivity, and enhanced baseline stability for formaldehyde detection.

In this work, a differential resonant photoacoustic cell (PAC) with an overall resonator length of 110 mm is designed and integrated with a 320 nm, 10 mW ultraviolet laser for online, real-time monitoring of H_2_CO. Compared with shorter resonator designs, the extended cavity provides improved acoustic confinement and enhanced buffer capacity, thereby suppressing flow-induced perturbations and improving signal stability. The differential PAC is fabricated by precision machining and adopts an integrated architecture consisting of two differential acoustic channels, two enlarged buffer chambers, and a single gas inlet and outlet. Despite the increased resonator length, the system maintains a compact and lightweight configuration, making it well suited for sensitive and stable trace-level H_2_CO detection under controlled laboratory conditions.

## 2. Materials and Methods

### 2.1. Photoacoustic Cell

When the PAC operates at its acoustic resonance, the resulting photoacoustic signal *S_PA_* can be expressed as

(1) $$S_{PA}=C_{PAC}\alpha PC$$

where *C_PAC_* is the cell constant, α is the absorption coefficient of the target gas, *P* is the excitation laser power, and *C* is the gas concentration. The cell constant is given by

(2) $$C_{PAC}=\frac{\left(\gamma -1\right)Q_{j}}{\omega_{j}V_{c}}∭p_{j}^{*}\left(\vec{r}\right)g\left(\vec{r},\omega \right)dV$$

where γ is the heat-capacity ratio, *Q_j_* is the Q-factor of the resonant mode, *ω_j_* is the modulation frequency, *V_c_* is the PAC volume, and the integral represents the overlap between the acoustic pressure field and the optical beam distribution. This constant characterizes the acoustic amplification of the PAC and depends on its geometry, volume, acoustic Q-factor, and modulation conditions. Hence, designing a PAC with a large cell constant is essential for achieving high signal-to-noise performance.

**Figure 1:**
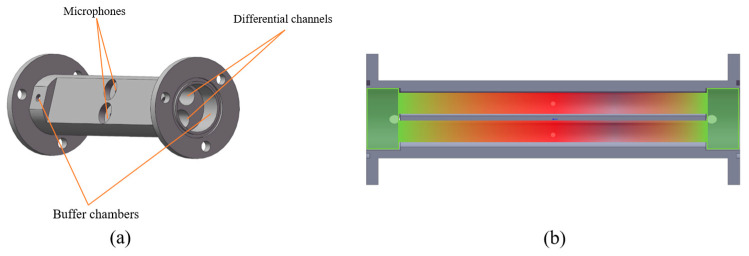
(**a**) Structure of the dual-cavity PAC with 110 mm cylindrical resonators and end buffer chambers. (**b**) Axial pressure profile in the PAC with a maximum at the cavity center and reduced pressure near the ends. Red color indicates the maximum signal, while green indicates the minimum signal.

In this work, a resonant photoacoustic cell (PAC) composed of two identical cylindrical acoustic cavities was fabricated by precision machining, as shown in Figure 1a. The PAC has an overall length of 110 mm and an overall width of 42 mm, corresponding to a footprint area of approximately 4620 mm^2^. Each cylindrical acoustic cavity has a radius of 4 mm, and both ends are connected to buffer chambers with a diameter of 20 mm and a length of 10 mm. The buffer chambers suppress flow-induced perturbations and reduce acoustic energy leakage at the boundaries, enabling the formation of stable longitudinal standing-wave fields. The overall geometry provides direct axial optical access and preserves the well-defined acoustic boundary conditions required for resonant photoacoustic excitation.

The acoustic behavior of the PAC was further examined using the experimentally obtained axial pressure-profile map, as presented in Figure 1b. In the designed 110 mm long differential photoacoustic cell (PAC), two microphones (Mic1 and Mic2) (EM258, Primo Microphones Inc., with a sensitivity of −32 ± 3 dB) are positioned at the longitudinal center of the two differential channels, respectively, for symmetric detection of the photoacoustic signals. The pressure distribution clearly reveals a longitudinal standing-wave pattern, with the central region of the resonator exhibiting the highest acoustic pressure (shown in red), corresponding to a pronounced pressure antinode. In contrast, the regions near both buffer chambers maintain significantly lower pressure levels (shown in green), indicating the formation of pressure nodes at the cavity boundaries. This pressure decay toward the buffer volumes reflects their acoustic compliance, which effectively increases the resonator’s acoustic length beyond its physical dimensions.

The spatial pressure pattern in Figure 1b confirms that the PAC supports a stable fundamental longitudinal mode under operating conditions. Such a distribution ensures efficient coupling of the modulated optical energy into acoustic energy, thereby enabling effective resonant amplification of the generated photoacoustic signal. The existence of a well-defined antinode at the cavity midpoint also validates the placement of the microphone and the optical focus, ensuring that the detection system operates at an optimal sensitivity point.

### 2.2. UV Laser Excitation Source

**Figure 2:**
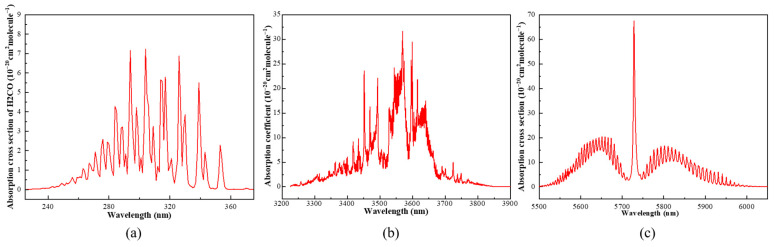
Partial absorption crosssection spectra of H_2_CO. (**a**) ultraviolet region from 240 to 360 nm. (**b**) infrared region from 3200 to 3900 nm. (**c**) infrared region from 5500 to 6000 nm.

The choice of excitation wavelength directly influences the sensitivity, selectivity, and long-term stability of photoacoustic spectroscopy. As shown in the absorption cross sections obtained from the HITRAN database in Figure 2a,c, H_2_CO exhibits continuous and structured absorption features in the 225–375 nm ultraviolet region, with particularly enhanced absorption between 280 and 330 nm. The absorption feature near 320 nm shows a stable line shape and moderate bandwidth, making it well suited for photoacoustic excitation. In contrast, the broadband absorption in the 3400–3600 nm mid-infrared region shown in Figure 2b, although relatively strong, is significantly broadened with a smooth spectral boundary, which is unfavorable for stable modulation and calibration. The 5700–5750 nm region in Figure 2c presents an even stronger but extremely narrow isolated absorption peak, requiring very high stability of the laser center wavelength and temperature; even a slight spectral drift can markedly reduce the absorption efficiency.

From an engineering perspective, narrow-linewidth mid-infrared light sources mainly rely on quantum cascade lasers (QCLs), whose structures are complex, highly temperature-sensitive, and limited in long-term operational stability. Moreover, maintaining narrow linewidth and power stability in compact systems is challenging. These engineering and cost constraints further limit the feasibility of employing mid-infrared high-absorption bands in portable or long-term online monitoring systems. Therefore, although the mid-infrared region offers stronger absorption, its practical implementation difficulty remains significantly higher than that of the ultraviolet region.

In comparison, the ultraviolet region not only provides more favorable spectral absorption characteristics but also allows the use of mature, stable, and low-cost laser sources to achieve compact and highly stable photoacoustic detection. Considering both absorption properties and system implementability, the 320 nm band offers a well-balanced compromise among sensitivity, selectivity, and long-term stability. Thus, this study selects 320 nm as the excitation wavelength for the UV-PAS system to achieve high-response and high-stability formaldehyde detection.

**Figure 3:**
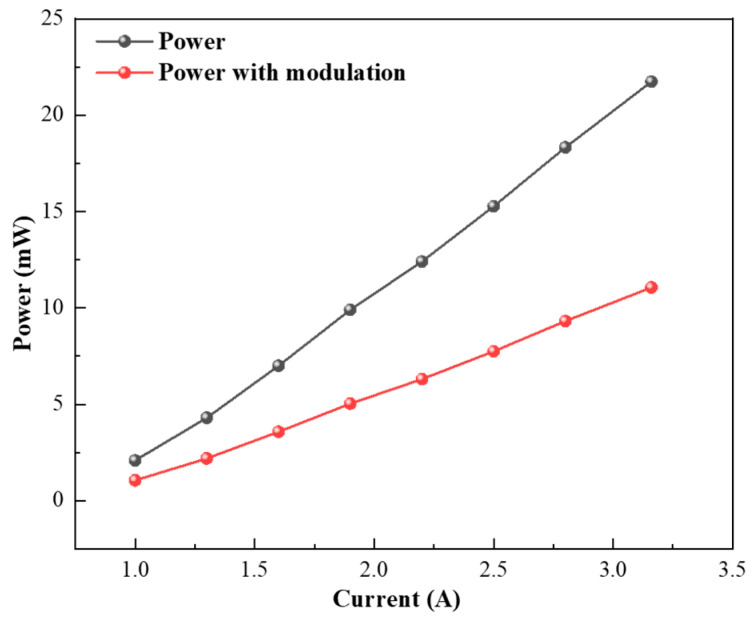
Transmitted laser power through the 110 mm PAC under unmodulated and chopper-modulated operation as a function of drive current.

To implement this strategy, a 320 nm, 10 mW UV laser (UV-FN-320–10 mW, China) is employed as the excitation source. The optical power transmitted through the 110 mm-long, 4 mm-radius PAC is measured under both continuous-wave (CW) operation and mechanical-chopper modulation, as shown in Figure 3. The transmitted laser power increases nearly linearly with drive current in both modes, reflecting the stable current–power characteristics of the UV diode source. At a drive current of 3.16 A, the transmitted power reaches 21.74 mW in the unmodulated CW state. After modulation by the mechanical chopper, the average optical power decreases to 11.06 mW, which is consistent with the 50% duty cycle of the chopper, indicating that the incident laser beam is periodically blocked for half of each modulation period and therefore delivers approximately half of the continuous-wave optical power into the PAC. The linearity and stability of the power–current response indicate that the excitation source provides reliable amplitude modulation, which is essential for maintaining a strong, reproducible photoacoustic signal under lock-in detection.

### 2.3. Experimental Setup

**Figure 4:**
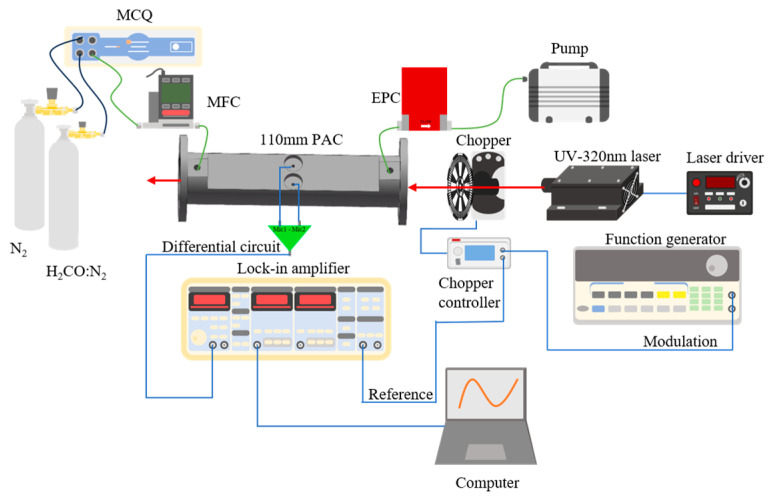
Schematic of the 320 nm UV-PAS system employing a 110 mm differential photoacoustic cell with buffer chambers.

The experimental configuration of the H_2_CO photoacoustic detection system is shown in Figure 4. A 320 nm, 10 mW ultraviolet laser is directed through a 110 mm-long photoacoustic cell featuring a large-volume cylindrical resonator with a radius of 4 mm. Compared with conventional compact PACs, the extended acoustic path length and enlarged resonator radius provide stronger acoustic confinement and reduced viscous–thermal losses, thereby enhancing photoacoustic conversion efficiency. Two cylindrical buffer chambers (20 mm in diameter and 10 mm in length) are integrated at both ends of the resonator to suppress flow-induced disturbances and minimize acoustic leakage. The cell also includes dedicated gas inlet and outlet ports that allow stable and controlled delivery of calibration mixtures.

Two identical electret condenser microphones are mounted symmetrically at the longitudinal midpoint of the enlarged resonator, corresponding to the pressure antinode of the fundamental acoustic mode. Their outputs are processed by a differential preamplifier to suppress common-mode disturbances such as environmental noise and flow fluctuations. Owing to the inherently strong acoustic field produced by the long, large-volume resonator, no additional internal structures are required to enhance the standing-wave amplitude.

Intensity modulation of the UV beam is achieved using a mechanical optical chopper (MC2000B, Thorlabs Inc., Newton, NJ, USA), driven by a TTL reference signal from a function generator (SPF05, SP Co., Chongqing, China) and operated at the resonant frequency of the photoacoustic cell to maximize excitation efficiency. The resulting photoacoustic signal is routed to a lock-in amplifier (SR830, Stanford Research Systems, Sunnyvale, CA, USA), where phase-sensitive demodulation is performed. The demodulated output is then transferred to a computer for real-time acquisition and analysis.

Calibration mixtures of adjustable H_2_CO concentration are prepared by diluting a 47.31 ppm H_2_CO:N_2_ standard gas with high-purity nitrogen using a gas distribution module (GB100, MCQ Instruments, Roma, Italy). The blended gas is introduced into the photoacoustic cell via a mass flow controller (MFC-500SCCM, China), enabling precise regulation of flow rate. Downstream of the cell, an electronic pressure controller (EPC100A-1X, China) maintains a stable internal pressure, and a diaphragm pump (N920G, KNF Co., Freiburg im Breisgau, Germany) exhausts the gas after measurement. Throughout the experiment, the flow rate and pressure inside the long, large-volume photoacoustic cell are independently regulated by the MFC and EPC, ensuring stable and reproducible operating conditions for photoacoustic detection.

## 3. Results

### 3.1. Photoacoustic Cell Resonant Frequency

**Figure 5:**
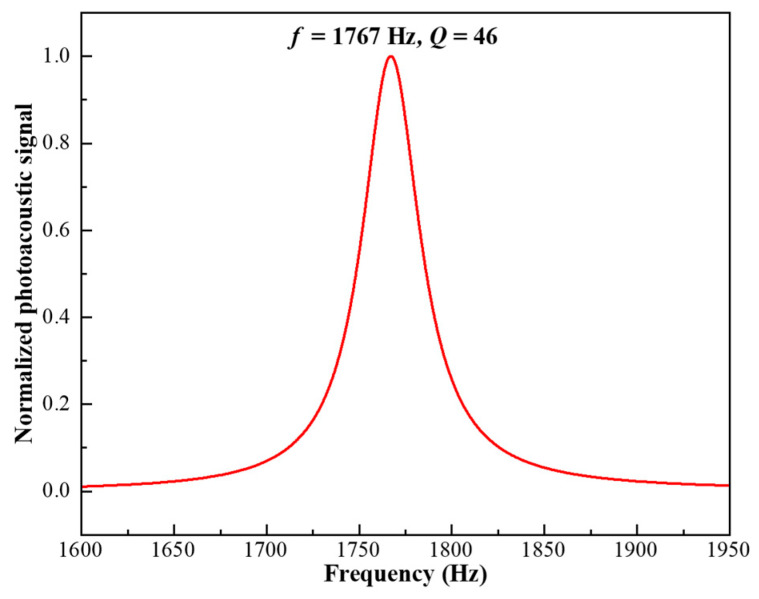
Normalized photoacoustic response of the resonant PAC under frequency sweep.

In photoacoustic spectroscopy, the modulation frequency of the excitation light must coincide with the acoustic resonance of the PAC to maximize signal generation. During all subsequent measurements, the laser modulation frequency was fixed at the resonance frequency and actively maintained without drift, ensuring stable and reproducible acoustic excitation throughout the experiments. To determine this resonance accurately, a frequency sweep from 1600 Hz to 1950 Hz with a resolution of 0.1 Hz was conducted using a 47.31 ppm H_2_CO:N_2_ mixture at a flow rate of 50 sccm and a pressure of 700 Torr. The resulting frequency response, shown in Figure 5, exhibits a well-defined resonance peak at 1767 Hz, characteristic of the fundamental longitudinal mode of a cylindrical resonator with partially compliant end boundaries. This sharp peak confirms the formation of a stable standing-wave field within the PAC.

The *Q*-factor of the PAC, calculated as the ratio of the resonant frequency to the full width at half maximum (FWHM), is 46, indicating strong acoustic confinement and low dissipative losses in the long, large-volume resonator. Based on this experimentally established resonance, the excitation beam is intensity-modulated at 1790 Hz, close to the resonance frequency, using a mechanical chopper driven at the same frequency. The resulting photoacoustic signal is demodulated by a lock-in amplifier operating in the 1f mode, ensuring optimal extraction of the modulated acoustic response.

### 3.2. Flow Rate Optimization

The modulated UV laser passes through the long-cavity PAC and excites H_2_CO molecules, generating periodic pressure waves that are detected by two microphones. Their outputs are processed through a differential amplifier, and the resulting photoacoustic signal is demodulated at the modulation frequency using a lock-in amplifier. A high-resolution data acquisition module records the demodulated voltage for subsequent analysis.

**Figure 6:**
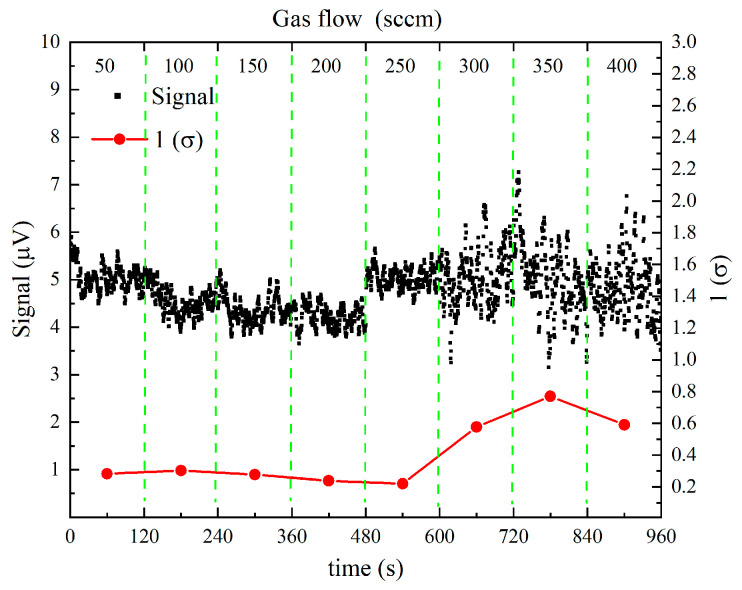
Photoacoustic signal amplitude and 1σ noise as a function of gas flow rate.

The dependence of the photoacoustic signal amplitude and noise behavior on the gas flow rate was investigated using a 47.31 ppm H_2_CO:N_2_ mixture at an integration time of 1 s, as illustrated in Figure 6. When the flow rate was increased from 50 sccm to 250 sccm, the photoacoustic signal amplitude remained essentially constant, indicating that the excitation efficiency and acoustic resonance were highly stable within this flow-rate range. However, as the flow rate was further increased from 250 sccm to 400 sccm, the signal amplitude began to exhibit more pronounced fluctuations, suggesting an increasing influence of flow-induced perturbations on the acoustic field.

In contrast, the noise level showed a clear dependence on the gas flow rate and reached a minimum at 250 sccm. This operating condition corresponds to the most stable acoustic environment within the long-cavity differential resonator, where flow-induced turbulence and external disturbances are effectively suppressed.

Notably, the ability to maintain a stable photoacoustic response up to a relatively high flow rate of 250 sccm represents a significant advantage over conventional PAS systems, which typically rely on low-flow operation to achieve acceptable noise performance. Owing to the optimized long resonator length, buffer chamber configuration, and differential detection scheme, the proposed UV-PAS system relaxes the stringent low-flow constraint and enables stable operation under elevated flow conditions. Therefore, a flow rate of 250 sccm was selected as the optimal operating point for subsequent measurements, providing a favorable balance between signal stability, noise suppression, and practical applicability for continuous online gas monitoring.

### 3.3. Pressure Optimization

Pressure is an important parameter in photoacoustic detection, as it simultaneously affects the acoustic characteristics of the 110 mm long resonant photoacoustic cell, the energy relaxation processes of H_2_CO molecules, and the efficiency of photoacoustic signal generation. To systematically evaluate the influence of pressure on detection performance, a 47.31 ppm H_2_CO:N_2_ gas mixture was introduced into the photoacoustic cell, and with an integration time of 1 s, the internal pressure was gradually increased from 200 Torr to 700 Torr. At each pressure condition, the photoacoustic signal was continuously recorded as a function of time to analyze the signal amplitude and its stability.

**Figure 7:**
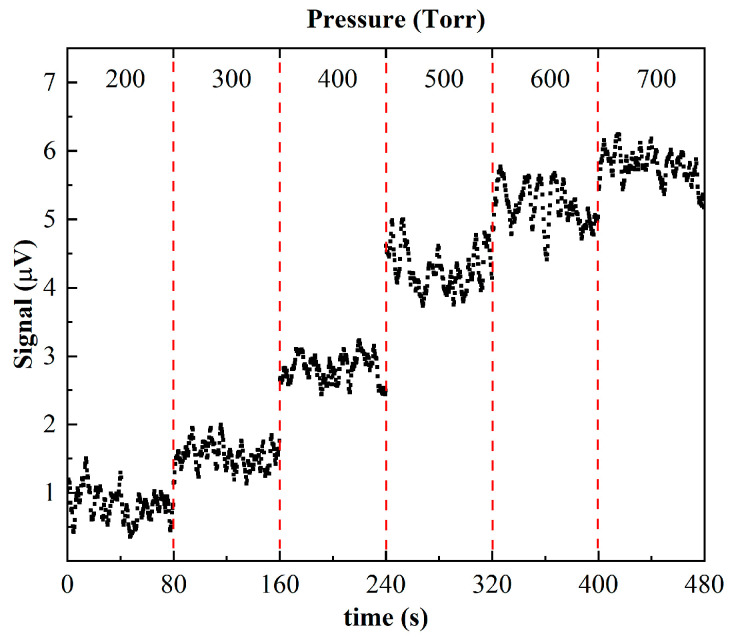
Photoacoustic signal at different pressures.

The average photoacoustic signal amplitude exhibits a clear stepwise increase as the pressure rises from 200 Torr to 700 Torr, as illustrated in Figure 7. Under low-pressure conditions (200–300 Torr), the signal amplitude is relatively small and remains at a low level. When the pressure is increased to 400 Torr and above, the signal amplitude is significantly enhanced; in particular, at 600 Torr and 700 Torr, the signal reaches its maximum and remains relatively stable. This behavior indicates that higher pressure promotes increased molecular collision frequency and more efficient energy transfer, thereby strengthening photoacoustic signal generation, while also contributing to a more stable acoustic field within the long-cavity resonator. Considering both signal strength and stability, the system shows optimal performance at 700 Torr. Accordingly, a pressure of 700 Torr is selected as the operating condition for subsequent photoacoustic measurements to achieve stronger signal output and more reliable detection results.

## 4. Discussion

**Figure 8:**
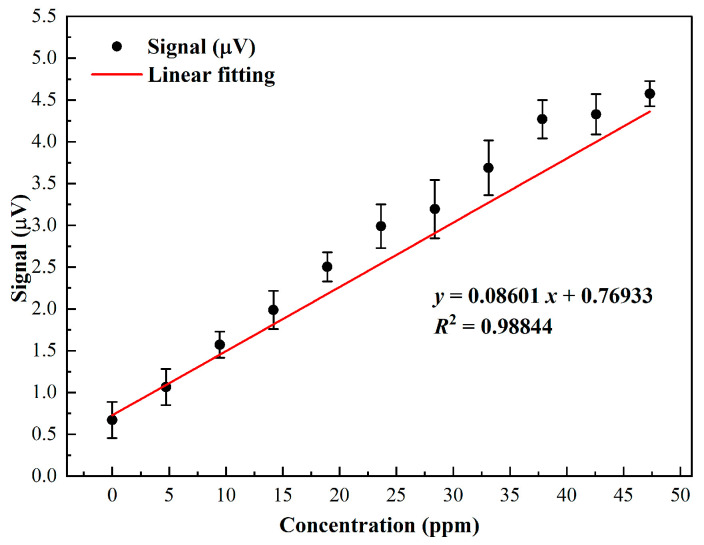
Linear dependence of the photoacoustic signal on H_2_CO concentration.

The detection sensitivity of the H_2_CO photoacoustic system was systematically evaluated by gradually increasing the H_2_CO concentration in H_2_CO:N_2_ mixtures. Gas mixtures with concentrations ranging from 4.73 ppm to 47.31 ppm were introduced into the 110 mm PAC, and the resulting photoacoustic signals were continuously recorded over a 200 s period with a 1 s averaging interval to ensure reliable measurement of both signal stability and amplitude. The experimental results are summarized in Figure 8, which combines both the temporal signal traces and the corresponding averaged amplitudes. For each concentration, the averaged amplitude was obtained from repeated measurements under identical conditions, and the corresponding standard deviation reflects the measurement repeatability and short-term stability of the system. The averaged amplitudes display a clear linear dependence on the H_2_CO concentration, with linear regression yielding an R^2^ value of 0.98844, confirming excellent linearity across the tested concentration range. When the PAC is filled with pure N_2_, the measured background noise level (1σ) is 0.21517 μV. Based on the slope of the linear fit (0.08601 μV/ppm), the detection limit of the system—calculated as the ratio of the background noise to the response slope—is approximately 2.50 ppm. These results demonstrate that the H_2_CO photoacoustic system provides reliable and quantitative detection over a sub-50 ppm range, with both high linearity and sufficient sensitivity for trace gas monitoring applications.

**Figure 9:**
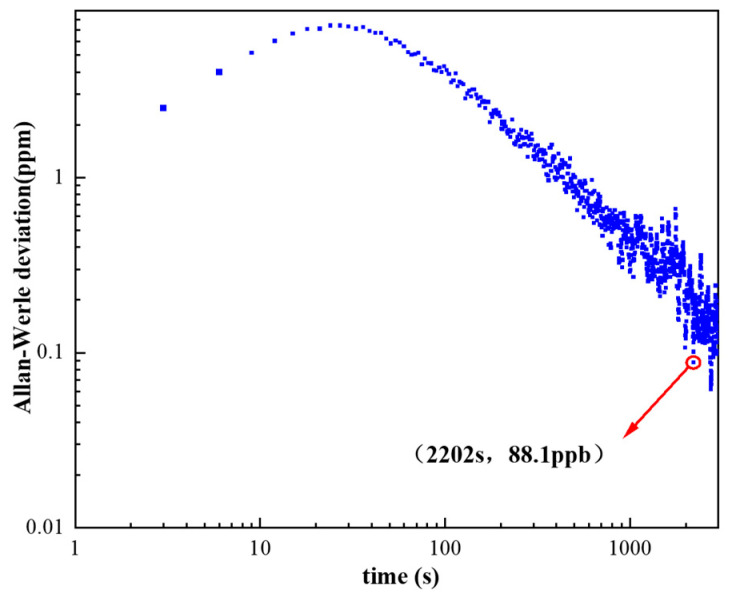
Allan–Werle deviation indicating ppb-level sensitivity for H_2_CO detection.

Allan–Werle deviation analysis was employed to evaluate the long-term stability and drift characteristics of the H_2_CO photoacoustic detection system. With pure N_2_ continuously flowing through the PAC, the photoacoustic signal was recorded over a two-hour interval, and the Allan–Werle deviation was calculated from the resulting time-domain voltage sequence. The computed deviation curve is presented in Figure 9.

The Allan–Werle deviation exhibits two distinct regions corresponding to different noise mechanisms. In the short-integration regime, the deviation initially increases, which reflects the reduction in measured PA signal amplitude caused by rapid baseline fluctuations and short-term technical noise (e.g., flow micro-disturbances, laser-power jitter, and electronic noise). As the integration time increases beyond this region, the deviation begins to decrease, indicating the dominance of white-noise averaging. In this regime, long-term averaging effectively suppresses uncorrelated fluctuations, leading to enhanced detection sensitivity. The transition between these regions marks the point at which drift and technical noise give way to statistically recoverable white noise.

As shown in Figure 9, with an integration time of 1 s, the deviation further decreases when the cumulative integration time reaches 2202 s, corresponding to an improved detection limit of 88.1 ppb. This result demonstrates that the 110 mm PAC–based UV photoacoustic system maintains stable long-term operation and can achieve sub-ppm sensitivity when appropriate averaging times are applied. The Allan–Werle analysis thus confirms that optimizing integration time is crucial for maximizing system performance, particularly under low-concentration or trace-gas detection conditions.

## 5. Conclusions

In summary, a nitrogen-background ultraviolet photoacoustic spectroscopy (UV-PAS) system for formaldehyde detection was designed, implemented, and evaluated. A precision-machined photoacoustic cell featuring a 110 mm long, 4 mm radius resonant cavity and dual buffer chambers was developed to enhance acoustic confinement and suppress flow-induced noise. Operating with a 320 nm UV excitation source and phase-sensitive detection, the system achieved a measured resonance frequency of 1767 Hz and a *Q*-factor of 46, enabling efficient acoustic amplification.

Systematic optimization of the flow rate and pressure identified operating conditions of 250 sccm and 700 Torr as providing the highest signal stability. Concentration-dependent measurements demonstrated a strong linear response, with an R^2^ value of 0.98844, yielding a minimum detection limit of 2.50 ppm at a 1 s integration time. The long-term stability of the system was evaluated using Allan–Werle deviation analysis. When the averaging time reached 2202 s with a time constant of 1 s, the detection limit was further improved to 88.1 ppb, indicating effective suppression of low-frequency drift.

## Data Availability

Some of the data are available upon request.
